# Wavelet image scattering based glaucoma detection

**DOI:** 10.1186/s42490-023-00067-5

**Published:** 2023-03-02

**Authors:** Hafeez Alani Agboola, Jesuloluwa Emmanuel Zaccheus

**Affiliations:** Afe Babalola University, Ado-Ekiti, Nigeria

**Keywords:** Wavelet scattering network, Scattering features, Glaucoma detection, Retinal fundus image

## Abstract

**Background:**

The ever-growing need for cheap, simple, fast, and accurate healthcare solutions spurred a lot of research activities which are aimed at the reliable deployment of artificial intelligence in the medical fields. However, this has proved to be a daunting task especially when looking to make automated diagnoses using biomedical image data. Biomedical image data have complex patterns which human experts find very hard to comprehend. Against this backdrop, we applied a representation or feature learning algorithm: Invariant Scattering Convolution Network or Wavelet scattering Network to retinal fundus images and studied the the efficacy of the automatically extracted features therefrom for glaucoma diagnosis/detection. The influence of wavelet scattering network parameter settings as well as 2-D channel image type on the detection correctness is also examined. Our work is a distinct departure from the usual method where wavelet transform is applied to pre-processed retinal fundus images and handcrafted features are extracted from the decomposition results. Here, the RIM-ONE DL image dataset was fed into a wavelet scattering network developed in the Matlab environment to achieve a stage-wise decomposition process called wavelet scattering of the retinal fundus images thereby, automatically learning features from the images. These features were then used to build simple and computationally cheap classification algorithms.

**Results:**

Maximum detection correctness of 98% was achieved on the held-out test set. Detection correctness is highly sensitive to scattering network parameter setting and 2-D channel image type.

**Conclusion:**

A superficial comparison of the classification results obtained from our work and those obtained using a convolutional neural network underscores the potentiality of the proposed method for glaucoma detection.

**Supplementary Information:**

The online version contains supplementary material available at 10.1186/s42490-023-00067-5.

## Introduction

Glaucoma constitute a weighty eye problem plaguing the human race. It is a group of diseases in which the optic nerve is impaired resulting in unalterable loss of vision. In majority of cases, this impairment is due to an increased intra ocular pressure within the eye. The ciliary body in the eye secretes a fluid known as aqueous humour into the space between the iris and the lens (i. e. the posterior chamber). The fluid then flows through the pupil into the space between the iris and the cornea (i. e. the anterior chamber) from where it drains through the trabecular meshwork, a spongy like structure at the base of the eye. A steady flow is established in a healthy eye as the rate of secretion balances the rate of drainage. In contrast, rate of drainage does not match the secretion rate in the unhealthy eye giving rise to accumulation of fluid in the anterior chamber. As accumulation increases, pressure builds up within the eye and the optic nerve which carries visual signals to the brain gets damaged leading to a permanent loss of vision. There are two main types of glaucoma. The open angle glaucoma and the angle closure glaucoma. In open angle glaucoma, the drainage canal (i.e. trabecular meshwork) is open while in angle closure glaucoma, the iris bulges forward and closes the drainage canal. In either case, the progression of the disease can be stopped with medical intervention but part of vision already lost cannot be restored even with surgery. This is why it is important to detect the signs of glaucoma early through regular eye examination particularly, eye pressure measurement. Although, the procedure carried out during a typical eye examination for glaucoma is fairly simple and non-invasive, the cost of the examination might constitute a barrier to regular and unlimited access to examinations most especially in the developing world. An important fact to note about glaucoma is that it can be present in patients with normal eye pressure and in like manner cause serious sight loss [24] . This fact proves that full understanding of this disease is still lacking. Therefore, many of its salient features, signs and manifestations are still left undiscovered. Many attempts have been made towards understanding the pathophysiology of glaucoma through mathematical modelling where more often than not, mechanical response of the optic nerve head is characterized as certain physiological parameters are varied. Prominent amongst these parameters are the intraocular pressure, cerebrospinal fluid pressure, scleral tension etc. However, little insights have only been drawn from the modelling exercise as glaucoma is dubbed multi-factorial disease thus developing a single model that will capture the essence of several factors such as ethnicity, diabetic status, gender, obesity, age etc. that contribute to glaucoma [25]has proved very difficult. The lack of good understanding of glaucoma might explain the reason why eyes with glaucomatuos field loss have high false negative responses during medical examinations [32]. Although we cannot rule out the influence of human error as fatigue, state of mind and carelessness may also contribute significantly to high false negative responses.

The associated issues with early detection and correct diagnosis of glaucoma raised in the foregoing can largely be tackled by considering automation of the glaucoma diagnosis process. Here, an intelligent system is envisaged. The system takes as input the retinal fundus image of a patient, performs a predefined mathematical operation on the image and produces an output encoding the health status of the eye. The predefined operation which is usually derived from techniques in biomedical image processing and machine learning determines the overall performance of the system. Several approaches focus on image segmentation methods including sparse dissimilarity constraint coding [10], super pixel classification [6, 9], and adaptive thresholding [18, 28] to delineate the optic disc and optic cup in retinal fundus images towards the estimation of features such as cup to disk ratio CDR [3], vertical cup and disc diameters (VCD and VDD) [23] and neuro-retinal rim (NRR) [15]. These features have been suggested [23] to have certain correlation to the presence of glaucoma. Another commonly explored approach is to process the entire retinal fundus image using time [21], frequency [22] or joint time-frequency analysis [20] in order to obtain new representation of the image data that are helpful for discriminatory or classification tasks. The use of Fourier analysis [13], Gabor transform [1] and wavelet transform [19] have been reported for glaucoma detection. Also, many classification algorithms that learn discriminatory features and classify data into different classes have been deployed [4, 34]. Specifically, in recent years, Convolutional Neural Networks (CNNs) [29] which are a class of Deep Neural Networks (DNNs) have gained tremendous traction in glaucoma detection research. This is largely due to the fact that they don’t require manual feature extraction as they are designed in a way that allows them to automatically obtain good data representation for classification task, learn the useful discriminatory features in the representation and finally classify the data into different classes [36]. Another reason is that CNNs have consistently displayed superior performance in several other time series [39] and image data [16] classification tasks. Although deep learning algorithms have been successfully used for glaucoma detection task [29], the computational resources required for their implementation is usually very huge. This stems from the fact that they usually have lots of tunable hyperparameters that can only be tuned properly with large amount of training data which is not always readily available in biomedical data analysis problems [12]. Secondly, their optimal network architecture and hyperparameters configuration are not well understood [27]. Therefore, a simpler alternative method that will produce the same or even better result than CNNs will be desirable.

Wavelet Image Scattering Network (WISN) is a representation or feature learning scheme described as very computationally cheap, well understood and very efficient in learning desirable data representation or automatic feature extraction (i.e. within class low-variance and between class high-variance features) [7]. The learned features can then be used downstream to build very simple and computationally cheap classification algorithms. Convolution, nonlinearity and pooling are the major operations carried out in the upstream section of all CNN architectures, these same operations are carried out efficiently in WISN by the successive application of wavelet filters, modulus operator and scaling filters respectively to the data. An important distinction between WISN and CNNs is that filters weights are learned in CNNs while they are fixed in WISN. As we write, we are not aware of any research work documenting a detailed performance of wavelet scattering features from retinal fundus image data on glaucoma detection. We note that wavelet scattering features were used in [17] however, the study is conspicuously silent on the effect of scattering transform parameters. Therefore, as far as we know, this paper presents the first research work studying the influence of wavelet image scattering network parameters on the scattering features obtained from retinal fundus image data for glaucoma detection. The aim is to study the potentiality of WISN as a reliable feature learning scheme for an envisioned fully automated glaucoma detection/diagnosis system. The rest of the paper is arranged as follows. Section Wavelet Scattering gives a concise theoretical background to wavelet scattering. Materials and methods are described in section Materials and Methods while the results and discussion are contained in sections Results and Discussion respectively. Lastly, we give our conclusion in section Conclusion.

## Wavelet Scattering

In this section, we provide a brief description of the theory behind 2-D wavelet scattering transform or wavelet image scattering. The advantage of using wavelets for biomedical signals analysis is discussed in section Biomedical Signal Analysis followed by the definition of the 2-D wavelet transform in section 2-D Wavelet Transform. Section Wavelet Scattering Network explains how wavelet scattering transform is derived from wavelet transform. A more detailed treatment of wavelet scattering transform can be found in [7].

### Biomedical Signal Analysis

Biomedical data or signals frequently exhibit slowly changing trends or oscillations punctuated with transients [37]. In particular, biomedical images usually consist of smooth regions interrupted by edges or abrupt changes in contrast. Generally, these abrupt changes are the most interesting part of the data both perceptually and in terms of the information they provide. The canonical Fourier Transform is a powerful signal analysis tool however, it does not represent abrupt changes in signals efficiently [35] in that it represents signals as a sum of sine waves which are not localized in time and space. In contrast, wavelet transform represents signals as sum of wavelets which are well localized in time and space [35]. This makes wavelet transform suitable for the analysis of most real world signals. The 2-D wavelet transform supplies the basic theory for wavelet scattering transform network to learn discriminatory features from image data.

### 2-D Wavelet Transform

A 2-D wavelet transform of an image signal $f(\overrightarrow{x})$, $f\in \ell ^2(\Re ^2)$ (i.e. finite energy signal) is given as

1 $$\begin{aligned} c(\overrightarrow{b}, a, \theta )= \int \psi \left( a^{-1}, r_{-\theta }\left( \overrightarrow{x}-\overrightarrow{b}\right) \right) f(\overrightarrow{x}) d\overrightarrow{x} \end{aligned}$$

Equation (1) defines a convolution operation $f\otimes \psi$ where $\psi$, the analysing wavelet or convolution kernel is dilated by $a>0$, translated by $\overrightarrow{b}\in \Re ^2$ and rotated by angle $\theta$ ($r_{-\theta }$ denotes the rotation operator). The analysing wavelet $\psi$ satisfies the admissibility condition which in most applications may be interpreted as $\psi$ having a zero mean (i.e. $\int \psi (\overrightarrow{x})d\overrightarrow{x} = 0$). When the admissibility condition is coupled with the localization capability of $\psi$ and its Fourier transform (i.e. band pass characteristic in the frequency domain) it becomes obvious that wavelet transform implements a local filtering in space ($\overrightarrow{x}$) and scale (*a*). This local filtering operates in constant relative bandwidth, $\Delta \omega /\omega$. Consequently, wavelet transform is more efficient at small scales or high frequencies particularly, in scanning singularities (transients or high frequency components in signals). The low-frequency components of the signal not captured are then decomposed through a separate function known as the scaling function ($\phi$) whose partial derivatives result in the analysing mother wavelet functions [33]. Even though wavelet transform is good at scanning localized features in signals, it suffers from the fact that it is translation covariant [14]. This means similar signals at dissimilar locations get mapped into separate signal classes, and thus making learning of discriminatory signal features by classification algorithms more complicated. Direct application of engineered features obtained directly from 2-D wavelet transform of retinal fundus images for glaucoma detection can be found in [26, 31]

### Wavelet Scattering Network

Fortunately, It can be shown [7] that the modulus of a wavelet transform, $\vert {f\otimes \psi }\vert$ makes it translation invariant. By taking this idea further, [7] created the scattering propagator ($V_p$), a path-ranked iteration operator on the modulus and applied the scaling function ($\phi$) to the propagator in order to capture the slowly varying features in the signal. Thus, introducing what they call the Wavelet scattering transform ($S_pf$)

2 $$\begin{aligned} V_pf=\vert {\vert {\vert {f\otimes \psi _1}\vert \otimes \psi _2}\vert \cdots \otimes \psi _m}\vert \end{aligned}$$

3 $$\begin{aligned} S_pf=\vert {\vert {\vert {f\otimes \psi _1}\vert \otimes \psi _2}\vert \cdots \otimes \psi _m}\vert \otimes \phi \end{aligned}$$

where $1, 2,\ldots , m$ denote the scattering stages. A wavelet image scattering network is produced by continued stage-wise evaluation of the convolution operation in Eq. (3). There are many mother wavelet functions in literature however, the choice of the analysing wavelet for a particular problem depends on the nature of the problem. Since we aim to detect relevant information such as segments and edges in biomedical images, anisotropic or directional wavelets are preferred. These wavelets are sensitive to rotations or directions and as such they can track oriented features such as segments and edges in images. Specifically, we chose the Morlet wavelet as the analysing wavelet in this work because it is the most widely used anisotropic wavelet in literature. The Morlet wavelet is obtained by tapering a sine wave by a Gaussian as shown in Eq. (4).

4 $$\begin{aligned} \psi _m = \exp (i2\pi {ft})\exp (-t^2/(2\sigma ^2)) \end{aligned}$$

## Materials and Methods

**Fig. 1:**
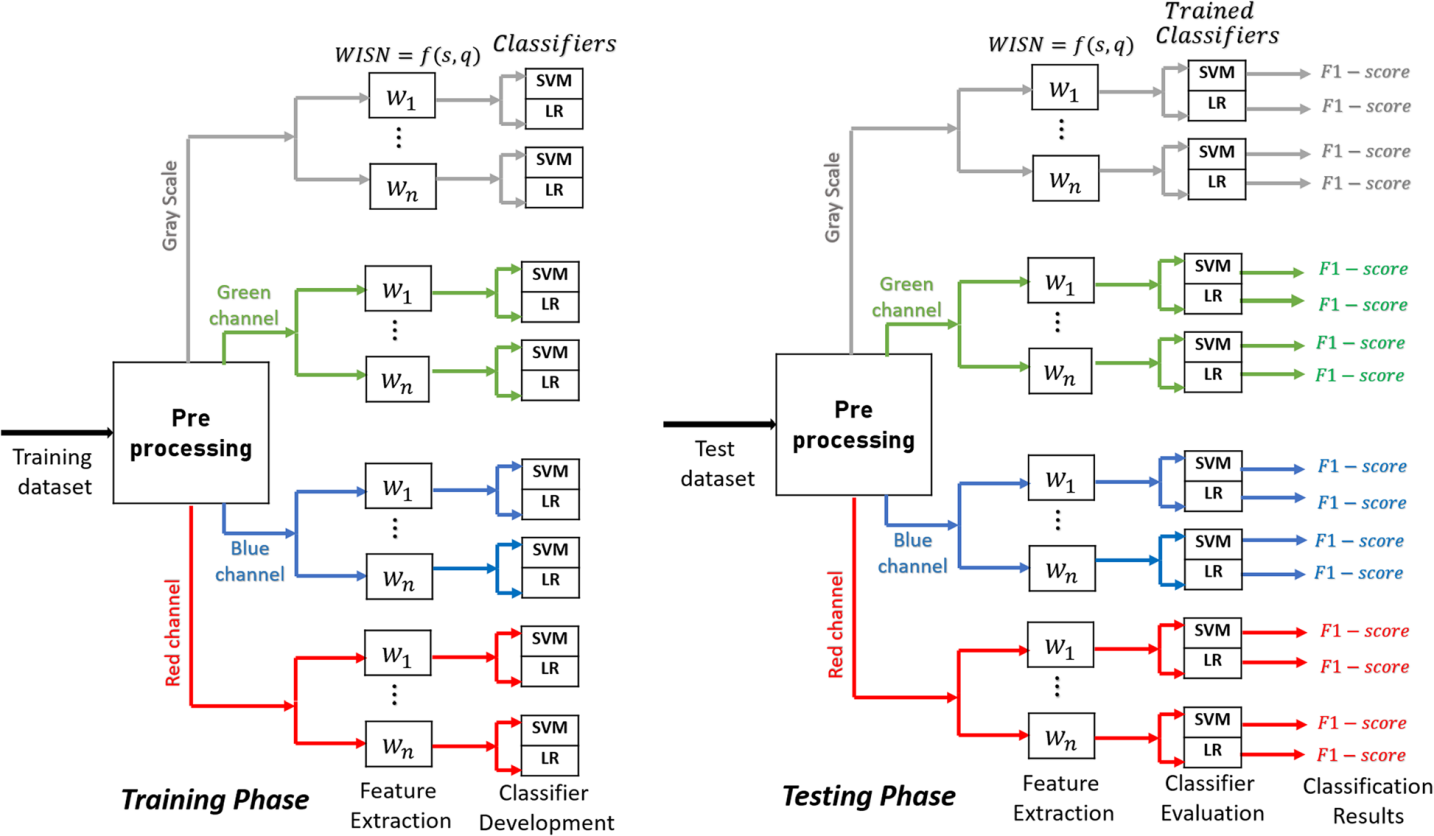
Training and testing workflows for the proposed method

This section presents detailed explanation on the methods and materials used in this work. The work-flow is depicted in Fig. 1.

### Data Acquisition

**Fig. 2:**
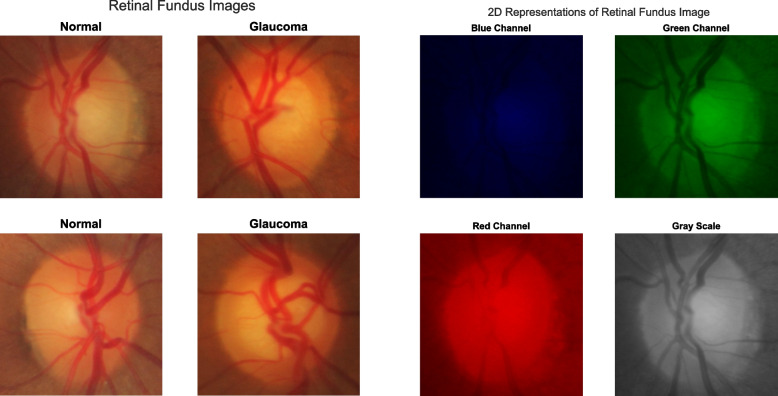
Retinal fundus images (left panel) and 2-D channel representations of a retinal fundus image (right panel)

The RIM-ONE DL image dataset [5] was downloaded and used for the study. Three hundred and thirteen and one hundred and seventy-two 3-D retinographies from healthy and glaucoma patients respectively are contained in the dataset. The images were captured in PNG format from subjects in three different Spanish hospitals. Additionally, the dataset is partitioned into training and test sets in two different ways: random partitioning and partitioning by hospital. In the former, the training and test sets are assembled randomly from all images in the dataset. However, in the latter, the partitioning was carried out with respect to hospital. That is, images taken in one hospital are used for the training set while those taken in the other two hospitals are used for the test set. The number of retinal fundus images in the training/test set of the randomly partitioned dataset is 339/146 out of which 219/94 images belong to the healthy or normal class while 120/52 belong to the glaucoma class. Similarly, the number of retinal fundus images in the training/test set of the hospital partitioned dataset is 311/174 out of which 195/118 images belong to the normal class and 116/56 images belong to the glaucoma class. The dataset is available for free download at https://bit.ly/rim-one-dl-images. Figure 2 shows retinal fundus images randomly selected from healthy and glaucoma classes in the dataset.

### Data Preprocessing

To be amenable to the 2-D wavelet transform, the 3-D images needed to be translated into their 2-D versions. Consequently, individual 2-D channels that is red, green and blue channels were extracted from all images. In addition, all images were converted to the 2-D gray scale format. Therefore, each image in the dataset has four different 2-D representations. These are blue channel (BC), green channel (GC), red channel (RC), and gray scale (GS) representations. Lastly, the 2-D images were resized to 300-by-300 in order to have a uniform image size as sizes of the original images vary significantly. All preprocessing steps were carried out in the Matlab environment using the Image Processing Toolbox. Representations for randomly selected images from normal and glaucoma image classes are displayed in Fig. 2.

### Development of our Wavelet Image Scattering Network

The wavelet scattering framework used for the wavelet image scattering decomposition was implemented in Matlab using the Image Processing and Wavelet toolboxes. The framework uses 2 complex-valued 2-D Morlet filter banks (i.e. 2 scattering stages). The scattering decomposition result depends on parameter setting in the framework. The parameters include Quality Factors (q), Invariance Scale (s) and Number of Rotations (r). In order to determine how sensitive the scattering features are to changes in individual parameter values, a t-test was conducted. The test gave information regarding the significance of difference in means of a particular feature as a given parameter is varied. For most of the features, there was a significant difference in the means when the scale invariance and quality factor parameters were varied. However, none of the features have significance difference in means with changes in the number of rotation parameter. Therefore, only the scale invariance and quality factors parameters were varied in the scattering framework. Quality Factors control the number of wavelets per octave in each of the filter banks. Although the flexibility of allowing wavelets within each octave may be desirable as it supports fine scale analysis, it could also escalate the computational complexity of the framework if many wavelets are used. Therefore, a balance must be struck somewhere in between. A maximum of four and three wavelets per octave in the first and second filter banks respectively was experimented in this study. The Invariance Scale parameter determines the spatial support of the scaling and wavelet filters (i.e. the spatial support of the scaling and wavelet filters can only take on values not exceeding the one specified for the invariance scale parameter). The default Invariance Scale parameter value in the framework is one-half the lower of the number of rows and columns in the image rounded to the nearest whole number. Therefore, in our case the default value for the Invariance Scale parameter is 150. As this default value does not imply the optimal value for the parameter, we experimented Invariance Scale parameter values in the range [25 150] with an incremental step of 25 (i.e. 25, 50, 75, 100, 125 and 150). Finally, the Number of Rotations parameter sets the number of rotations of each wavelet in each filter bank in the scattering framework. As stated earlier, varying this parameter showed an insignificant effect on the scattering features. Therefore, six clockwise rotations (with linearly spaced angles between $0$ and $\pi$ radians) per wavelet per filter bank which corresponds to the framework default parameter setting was used in the study.

### Wavelet Scattering Learned Features

Regardless of the WISN parameter configuration used, the dimension of the resulting feature space for each retinal fundus image is $x \times y \times z$. That is there are $x$ scattering paths and individual scattering path gives a scattering coefficient matrix of dimension $y \times z$. Subsequently, we obtained the mean along the 2nd and 3rd dimensions (i.e. $y$ and $z$ respectively) of the scattering coefficient matrix to arrive at $x$ - element feature vector for individual image in the training and test datasets. This resulted in a significant data reduction from 90,000 (i.e. $300 \times 300$ image size) elements to $x$ where $x$ varies between 100 and 700 in this study.

### Design of Classification Algorithms

The workflow for the classifier design include training and hyperparameter optimization. The classification algorithms explored are the binomial Logistic Regression (LR) and binary Support Vector Machine (SVM). To serve as a check, a simple Convolutional Neural Network (CNN) classifier was also considered. The CNN was constructed to have a convolution layer with 25 10-by-10 filters with 1-by-1 strides. This is followed by a RELU activation, max pooling layer and a fully connected layer. Furthermore, a softmax layer was deployed in order to normalize the output of the fully connected layer into probabilities. Lastly, a cross entropy loss was used as the loss function. Each classifier type was designed using individual 2-D image data representation and feature vectors from the training datasets were used exclusively for training and optimizing the classifiers. Optimized hyperparameters include regularization penalty and learning rate for LR and Kernel function, Kernel scale and box constraint for SVM. A 5 - fold cross validation scheme was used for training the LR/SVM classifiers. The best hyperparameter setting was the one that returned the minimum cross-validated classification loss for the LR/SVM model and was eventually used for classifying the test sets. The Bayesian optimization procedure was used and it was implemented in Matlab.

### Performance Metric

Since there is an imbalance in the number of samples in the two data classes (see section Data Acquisition), the appropriate performance metrics for classifier evaluation is the F1-score. F1-score describes a classifier’s performance on individual data class. To obtain the F1-score, one needs to first calculate precision and recall for the classifier. Precision measures the proportion of correct positive predictions in all positive predictions while recall measures the proportion of correct positive predictions in all positive data samples. In other words, precision answers the question: out of all positive predictions made by the classifier how many are truly positive? However, recall answers the question: out of all positive data samples how many are classified as positive? The harmonic mean of precision and recall is the F1-score. That is the F1-score reports the classifier’s performance in terms of both precision and recall. It is worthy of note that precision and recall are both defined in terms of relevance, meaning that they are defined relative to what the experimenter designates as the positive data class. Here, we refer to the glaucoma class is the positive class.

**Fig. 3:**
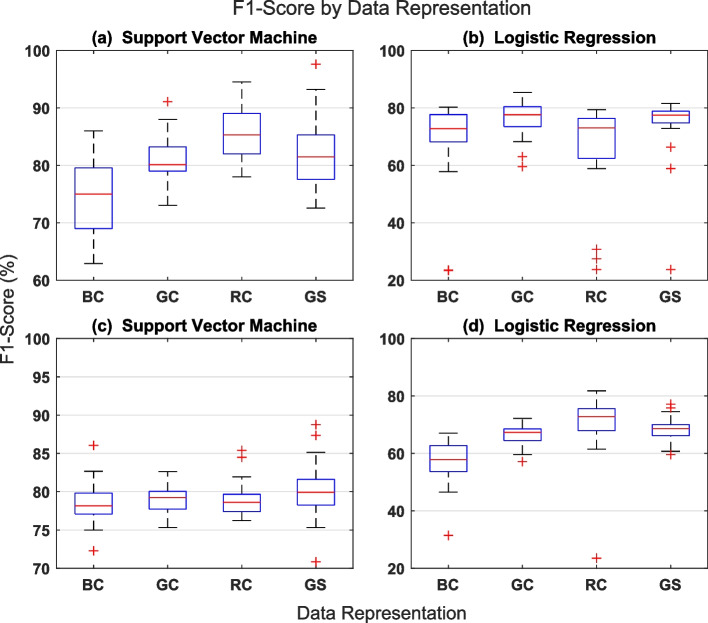
Boxplots summarizing the influence of WISN parameter setting on classification result for data partitioning by hospital (lower panel) and random partitioning (upper panel) schemes

**Fig. 4:**
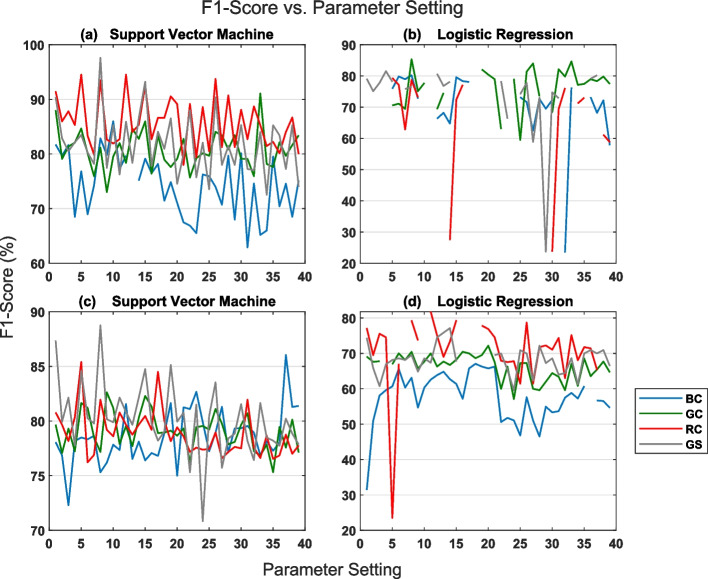
Plots of F1-score against parameter setting for data partitioning by hospital (lower panel) and random partitioning (upper panel) schemes

## Results

We present the results of the retina fundus image classification experiments in the boxplots shown in Fig. 3. Classification results for the test set in the case of data partitioning by hospital are displayed in Fig. 3c and d while those for random partitioning are displayed in Fig. 3a and b. The boxplots summarizes the influence of WISN parameter setting, data representation scheme and classification algorithm type on the classification accuracy of the test sets. Results in Fig. 3a and c were obtained using support vector machine classifier while those in Fig. 3b and d were obtained using logistic regression classifier. Each boxplot within a Figure reveals the distribution of the classification accuracy results and hence the effect of WISN parameters setting on the classification accuracy results for each data representation scheme (i.e. BC, GC, RC, and GS). The influence of WISN parameter setting on F1-score is shown more explicitly in Fig. 4 where we have plotted F1-scores against individual parameter configuration for each of the 2-D channel representations and classification algorithm. There are thirty-nine (39) distinct parameter settings explored in this work (see section Development of our Wavelet Image Scattering Network). In Table 1 we have shown specifically, the best set of hyperparameter values for each of the classifiers with respect to individual 2-D channel representations and data partitioning scheme. The best set of hyperparameter values are those that produced the maximum F1-scores. Lastly, in Table 2 we have compared the classification results obtained from our proposed method (i.e. combination of WISN features and simple classifiers) and an ordinary convolutional neural network (CNN) classifier (section Design of Classification Algorithms) for both data partitioning schemes.

## Discussion

Generally, it is observed from Fig. 3 that highest F1-scores were obtained by the support vector machine classifier in both data partitioning schemes. For the random partitioning, highest F1-score of 98%/85% was achieved by the support vector machine/logistic regression classifier on the gray scale/green channel data representation. For the partitioning by hospital however, F1-score of 89%/83% was achieved by the support vector machine/logistic regression classifier on the gray scale/red channel data representation. It is quite reasonable to expect that the highest F1-score value would come from the random partitioning dataset. In the random partitioning scheme, there are chances that some subjects would have their data placed in both the training and test datasets. As patient-specific features have been found to be present in glaucoma [38], learning algorithms can pick up these features during training and this will definitely make them perform well during testing. Conversely, in the partitioning by hospital scheme, the training and test datasets contain data from different subjects.

Furthermore, F1-score is highly sensitive to WISN parameter setting for each of the 2-D channel representations and classification algorithms. This is clearly highlighted by Fig. 4. Figure 4 further reveals the superiority of support vector machine to logistic regression in correctly classifying the test datasets. It is observed that there are a number of missing F1-score values in the plots. This is more pronounced in the plots for logistic regression where all the data representations (gray level, red, blue and green channels) have missing F1-score values. Missing F1-score values occur as a result of NANs. NaNs are in turn occasioned by the occurrence of zero positive predictions by the classification algorithm at a particular hyperparameters setting. This means that the classification algorithm failed to classify none of the retinal fundus images in the glaucoma class as positive when certain hyperparameters values are used. This set of hyperparameters value is the worst for the classification algorithm. Table 1 clearly shows that a scale invariance of 125 and quality factor [1,1] (i.e. one wavelet per octave in each of the filter banks) which corresponds to hyperparameter setting number 8 (see Fig. 4) appears to be the best hyperparameter setting out of the thirty-nine (39) explored in the classification problem. Furthermore, the gray scale representation proved to contain the most discriminatory features between healthy (normal) and glaucomatuous retinal fundus images amongst the four 2-D channel representations. The gray scale representation gave the best F1-score result in the two data partitioning schemes.

As evident from Table 2, the new method proved to be a better alternative to CNN, at least for this problem and the particular CNN configuration used. For the random partitioning scheme, the CNN trailed both the support vector machine and logistic regression classifiers. However, the CNN trailed only the support vector machine classifier but led the logistic regression classifier in the data partitioning by hospital scheme. The best 2-D channel representation for the CNN in both cases is the red channel.

Regarding ease of algorithmic implementation, an examination of the inference time was conducted to access the ease of implementation of our method. Five batches of 25 retinal fundus images were drawn randomly from each class for the inference analysis. The inference time (in milliseconds) is the average of the time taken to classify all 25 images in each batch. We used a CPU device with 32 Gigabyte of memory and Intel Core i9 (8cores) execution unit. The inference time included the time it took to read the re-sized 2D channel images from file, obtain the wavelet scattering features and classify the images. The inference time for each scheme explored is shown in Table 3. The wavelet scattering network schemes have the least inference time and the scheme with the support vector machine is about 2 times faster than that with logistic regression.

**Table 1:** Best hyperparameter setting

Data		WISN		
Partitioning	Classification	Parameter	Image	F1-score
Scheme	Algorithm	Setting	Representation	(%)
Random	Support	$s = 125$	Gray Scale	98
	Vector	$q = [1,1]$		
	Machine			
	Logistic	$s = 125$	Green Channel	85
	Regression	$q = [1,1]$		
Hospital	Support	$s = 125$	Gray Scale	89
	Vector	$q = [1,1]$		
	Machine			
	Logistic	$s = 125$	Red Channel	82
	Regression	$q = [3,2]$		

Finally, we compare our results with those reported in literature. The RIM-ONE DL (RIM-ONE for Deep Learning) dataset was released in 2020 as a refined version of the three initially released RIM ONE datasets (RIM-ONE v1, v2, and v3). The dataset was specifically optimized for deep learning applications [5]. Different CNN architectures have been utilized for the classification of both hospital and random partitioned datasets. Table 4 compares the classification accuracy results from these CNN architectures and our method. To the best of our efforts, only one publication [17] was found in literature to have applied wavelet scattering network features for glaucoma detection and the dataset used in the article is the initial dataset (i.e. RIM-ONE v3). The results from the work is also included in Table 4. This indicates that our work is the first to apply wavelet scattering features for glaucoma detection using the RIM-ONE DL dataset. From the table, it is obvious that our method gave better accuracy values in both hospital and random partitioned datasets.

**Table 2:** Classification accuracy results for SVM, LR and CNN

Data			WISN	Maximum
Partitioning	Classification	Image	Parameter	F1-score
Scheme	Algorithm	Representation	Setting	(%)
Random	Support	Gray Scale	$s = 125, q = [1, 1]$#8	98
	Vector	Red Channel	$s = 100, q = [4, 1]$#5	94
	Machine	Green Channel	$s = 75, q = [1, 1]$#33	91
		Blue Channel	$s = 125, q = [3, 1]$#10	82
	Logistic	Green Channel	$s = 125, q = [1, 1]$#8	85
	Regression	Gray Scale	$s = 100, q = [3, 2]$#4	82
		Blue Channel	$s = 125, q = [1, 1]$#8	80
		Red Channel	$s = 125, q = [4, 1]$#12	79
	Convolutional	NA	Red Channel	82
	Neural			
	Network			
Hospital	Support	Gray Scale	$s = 125, q = [1, 1]$#8	89
	Vector	Blue Channel	$s = 125, q = [4, 1]$#37	86
	Machine	Red Channel	$s = 100, q = [4, 1]$#5	85
		Green Channel	$s = 125, q = [2, 1]$#9	83
	Logistic	Red Channel	$s = 125, q = [3, 2]$#11	82
	Regression	Gray Scale	$s = 125, q = [4, 3]$#14	77
		Green Channel	$s = 150, q = [4, 2]$#31	72
		Blue Channel	$s = 150, q = [3, 2]$#29	67
	Convolutional	NA	Blue Channel	83
	Neural			
	Network			

**Table 3:** Inference Time (per image) comparison

Model	Time (ms)
WISN + SVM	894.55
WISN + LR	1562.06
CNN	1725.21

## Conclusion

This work exploits wavelet image scattering to obtain within class low-variance representations from 2-D channel representations of retinal fundus images for glaucoma detection. Utilizing the 2-D scattering transform with fixed filter weights and simple classification algorithms, we were able to attain a maximum of 98% and 89% correct classification on a held-out test sets. Wavelet Image Scattering Network proved to be a robust and effective feature extractor for glaucoma detection requiring only a minimal set of user-specified parameter values. On the same problem but with a simple Convolutional Neural Network whose filters were learned, we achieved a maximum of 82% and 83% correct classification. It is important to reiterate that our work is not intended as an absolute comparison of WISN and CNNs but rather, to demonstrate the potentiality of Wavelet Image Scattering for producing robust and efficient representations of retinal fundus image data for glaucoma detection learning task. WISN and CNNs have different hyperparameter and architectural changes that can significantly influence the classification results. For instance, highly optimized CNN architectures such as VGG19 [8], VGG16 [30], Xception [11], and ResNet50 [2] have been reported to have achieved 93%, 92%, 91% and 91% correct classification respectively on the random data partitioning test set and 85%, 85%, 84% and 79% respectively on the data partitioning by hospital test set. A future research task of our group is to apply our method to all publicly available glaucoma fundus datasets while also exploring different retina fundus image pre-processing modalities. The results from such task will further give insight into the versatility and reliability of our method.

**Table 4:** Wavelet scattering network versus CNN architectures on RIM-ONE DL dataset. Left: Randomly partitioned, Right: Partitioned by hospital. Accuracy values for the CNN Architectures were copied from https://github.com/miag-ull/rim-one-dl

Network	Accuracy(%)	Accuracy(%)
VGG19	93	85
VGG16	92	85
Xception	91	79
ResNet50	91	83
MobileNetV2	90	53
DenseNet	90	78
MobileNet	93	82
InceptionResNetV2	91	76
InceptionV3	89	80
NASNetMoile	75	79
WISN (RIM-ONE DL)our work	98	89
WISN (RIM-ONE v3)[18]	93	NA

## Supplementary Information

**Additional file 1.** **Glaucoma Random**. This is an excel workbook containing classification results for the glaucoma class in the test set of the randomly partitioned dataset. The workbook contains three worksheets. Each worksheet contains results for individual classification algorithm (i.e. SVM, LR and CNN).

**Additional file 2.** **Glaucoma Hospital**. This is an excel workbook containing classification results for the glaucoma class in the test set of the dataset partitioned by hospital. The workbook contains three worksheets. Each worksheet contains results for individual classification algorithm (i.e. SVM, LR and CNN).

**Additional file 3.** **Healthy Hospital**. This is an excel workbook containing classification results for the healthy or normal class in the test set of the dataset partitioned by hospital. The workbook contains three worksheets. Each worksheet contains results for individual classification algorithm (i.e. SVM, LR and CNN).

**Additional file 4.** **Healthy Random**. This is an excel workbook containing classification results for the healthy or normal class in the test set of the randomly partitioned dataset. The workbook contains three worksheets. Each worksheet contains results for individual classification algorithm (i.e. SVM, LR and CNN).

**Additional file 5.** **Wavelet Image Scattering Paper Code**. This file contains the codes used for the project.

## Data Availability

Data generated during the work is available in the related information files. Furthermore, the dataset used for the study is available for free download at https://bit.ly/rim-one-dl-images. We have also made codes used for the project available at https://drive.google.com/file/d/1KjxdRHzeK6BK2uqHtCDaXc3ub7AXG8bb/view?usp=share_link. Any additional information relating to the work can be sought from the authors through reasonable request from the corresponding author.

## Abbreviations

BC: Blue channel
CDR: Cup to disc ratio
CNN: Convolutional Neural Network
DNN: Deep Neural Network
GC: Green Channel
GS: Gray Scale
LR: Logistic Regression
NRR: Neuro-Retina Rim
RC: Red Channel
RELU: Rectified Linear Unit
SVM: Support Vector Machine
VCD: Vertical Cup Diameter
VDD: Vertical Disc Diameter
WISN: Wavelet Image Scattering Network
